# Research on joint model relation extraction method based on entity mapping

**DOI:** 10.1371/journal.pone.0298974

**Published:** 2024-02-23

**Authors:** Hongmei Tang, Dixiongxiao Zhu, Wenzhong Tang, Shuai Wang, Yanyang Wang, Lihong Wang

**Affiliations:** 1 School of Computer Science and Engineering, Beihang University, Beijing, China; 2 School of Aeronautic Science and Engineering, Beihang University, Beijing, China; 3 Jiangxi Research Institute of Beihang University, Nanchan, China; 4 National Computer Network Emergency Response Technical Team/Coordination Center of China, Beijing, China; University of Kurdistan Hewler, IRAQ

## Abstract

Relationship Extraction (RE) is a central task in information extraction. The use of entity mapping to address complex scenarios with overlapping triples, such as CasRel, is gaining traction, yet faces challenges such as inadequate consideration of sentence continuity, sample imbalance and data noise. This research introduces an entity mapping-based method CasRelBLCF building on CasRel. The main contributions include: A joint decoder for the head entity, utilizing Bi-LSTM and CRF, integration of the Focal Loss function to tackle sample imbalance and a reinforcement learning-based noise reduction method for handling dataset noise. Experiments on relation extraction datasets indicate the superiority of the CasRelBLCF model and the enhancement on model’s performance of the noise reduction method.

## 1 Introduction

Relation Extraction (RE) is a critical task in information extraction. The task was formalized initially at the MUC-7 [1] conference in 1998. It is mainly aimed at categorizing the semantic relations between entities. This type of information is crucial for constructing semantic knowledge bases (KBs), which can be used to infer the relations between various entities further [2].

Currently, methods based on entity mapping for relationship extraction have demonstrated promising results in handling complex tasks involving overlapping triples [3]. Wang et al. [4] introduced the TPLinker, an end-to-end sequence labeling model based on a handshake labeling strategy. This approach stands as the first single-stage joint extraction model capable of extracting various overlapping relationships without being influenced by exposure bias. Sun et al. [5], recognizing the correlations between related tasks such as entity recognition and relationship extraction, devised a progressive multi-task learning model with explicit interaction (PMEI). They utilized early-predicted interactions to refine task-specific representations. Tian et al. [6] mapped the relationship triples of text into a three-dimensional space, modeling the cube from various perspectives (slice, projection, and contraction), and subsequently extracting the triples. Liang proposed [7] the SGNet, a joint extraction model based on Soft Pruning and GlobalPointer. By leveraging BERT pre-trained models to obtain text word vectors enriched with contextual information, the method then utilizes graph operations to derive both local and non-local information from the vectors, addressing the challenge of extracting overlapping triples. However, these methods still grapple with issues such as oversensitivity in identifying head entities (despite efforts to consider sentence continuity and enhance recognition accuracy), sample imbalances, a dearth of datasets, and the prevalent noise from automatically labeled remotely supervised methods.

The existential difficulties of the relation extraction task include the lack of labeled datasets, the difficulty of labeling, and ternary overlap. In this paper, we propose a joint extraction method for relations based on entity mapping, which improves on the CasRel [3] extraction model and focuses on improving the head entity extraction performance to alleviate the sample imbalance problem that exists in the model during tail entity decoding; to mitigate the noise impact on the model in the distant supervised dataset to effectively improve the accuracy of the relation extraction.

The main contributions of this paper are as follows:

(1)We Propose a joint extraction method based on entity mapping. First, a bidirectional extended short-term memory network BiLSTM+CRF sequence labeling model is designed as the head entity decoder to improve the head entity extraction performance. Then, Focal Loss is proposed as the loss function of the tail entity decoder to alleviate the imbalance of tail entity samples effectively.
(2)We propose a distant supervised noise reduction method based on reinforcement learning. Firstly, the deep reinforcement learning noise reduction agent is trained on the original distant supervision training set. Then, the trained noise reduction agent is used to identify all the erroneous positive instances in the original dataset. The positive instances are reallocated to the negative instances dataset portion, which removes the influence of noise on the accuracy of relation extraction and enhances the performance of the relation extraction model CasRelBLCF on distant supervision datasets.
(3)Experiments on publicly available relation extraction datasets show that the CasRelBLCF model performs better than the original model. Meanwhile, the experiments verify that our proposed distant supervised noise reduction method can effectively filter the false-positive noise of the distant supervised dataset and improve the training effect of the model.

The paper is organized as follows: Section 2 reviews and discusses related work. Section 3 presents the design details of the CasRelBLCF model proposed in this paper. Section 4 describes the experimental program, including the dataset, evaluation metrics and implementation details, quantitative and qualitative evaluation results, and analysis. Section 5 concludes.

## 2 Related work

Relationship extraction primarily comprises three approaches: rule-based matching, machine learning-based, and deep learning-based methods. Rule-based methods rely on expert-defined extraction rules, which entail high design costs and hinder scalability across diverse domains and relationship types [8]. Machine learning-based relationship extraction methods leverage statistical language models for training and have achieved superior results with reduced human intervention. For instance, methods based on maximum entropy models [9], CRF models [10], naive Bayes, and perceptron models [11] have been employed. Nonetheless, these approaches still necessitate expert-designed sentence features, and their generalization and extraction performance remain suboptimal. Deep learning-based relationship extraction algorithms utilize encoding layers, such as Convolutional Neural Networks (CNN) and Recurrent Neural Networks (RNN), to automatically extract sentence features. Generally, relationship extraction methods can be categorized into pipeline and joint learning approaches [12]. Pipeline methods predominantly employ neural network structures like CNN [13], RNN [14], graph convolutional networks [15, 16], reinforcement learning [17], and various encoders [18] for relationship extraction. This method separates entity recognition and relationship extraction into distinct tasks. While its advantage lies in its intuitive and clear structure, it suffers from error propagation issues and fails to enable the model to learn shared features between the two tasks.

Joint learning methods, based on shared parameter joint learning models, consider entity recognition and relationship extraction as a unified task. This approach effectively captures shared features between the two-stage tasks and has demonstrated notable success in relationship extraction. Zeng et al. [19] introduced a re-decodable relationship extraction model, which innovatively addressed the issue of overlapping triples. Liu et al. [20] proposed an end-to-end Binary Cross-labeling (BCT) scheme and developed a BCT framework to jointly extract overlapping entities and triples. Su et al. [21] presented an end-to-end neural framework based on a decomposition model, combining multi-granular relationship features to extract overlapping triples. The model exhibits significant advantages in extracting long-tail relationships. Yang et al. [22] introduced an attention mechanism to learn fine-grained sentence representations for different relationships, capturing bidirectional dependency relationships between subjects and objects. To further explore and leverage the correlations between semantic relationships, Chen [23] proposed a relationship-first detection model. This model first detects potential relationships in the sentence and then performs entity recognition for each specific relationship, avoiding additional computations for redundant relationships. Luo [24] presented a context-aware network for joint relationship extraction with a cross-task attention mechanism, utilizing semantic correlations between subtasks to enhance performance. Collectively, these studies address the challenge of overlapping triples from perspectives such as long-tail relationships, multi-granular relationship feature extraction, and semantic relationship correlations. Experimental results validate that joint learning can significantly enhance performance in relationship extraction tasks.

Remote supervision is a technique for automatically labeling relationship extraction datasets, reducing the dependency on manual annotation and facilitating easier model scalability across various domains. However, noise present in remote supervision datasets adversely affects model performance. To enhance model robustness, researchers have proposed leveraging remote supervision techniques for relationship extraction [25]. With advancements in deep learning technologies, segmentation convolutional neural networks [26], attention mechanisms [27], and unlabeled remote supervision [28] have also been applied to relationship extraction tasks based on remote supervision datasets. Ye et al. [29] not only considered intra-bag noise but also addressed inter-bag noise. In 2020, Xiao et al. [30] introduced the DocRE pre-trained model capable of capturing valuable information from noisy datasets. Subsequently, reinforcement learning began to play a role in noise reduction techniques. Reinforcement learning-based noise reduction models are mostly positioned at the beginning and end of relationship extraction models, without imposing specific requirements on the structure of the relationship extraction model. Therefore, they exhibit higher flexibility than previous noise reduction methods. Utilizing reinforcement learning techniques [31, 32] for noise reduction typically involves preprocessing and post-processing the model as a plug-in, effectively leveraging the information in noisy data and demonstrating excellent scalability. Han et al. [33] proposed a joint entity relationship extraction model (SMHS) based on a cross-level multi-head selection mechanism, transforming entity relationship extraction into a cross-level multi-head selection problem. Experimental results on the classic English dataset NYT and the publicly available Chinese relationship extraction dataset DuIE 2.0 demonstrated that this approach outperformed baseline methods.

## 3 Proposed model

### 3.1 Problem analysis

In the field of relation extraction, how well the overlapping ternary problem is solved determines the extraction performance of the relation extraction model to a certain extent. Overlapping ternary refers to a sentence in which the same entity participates in multiple relations, and most of the past relation extraction models need to be stronger on the overlapping ternary problem. In 2020, the ACL conference proposed an entity mapping-based relation extraction model CasRel [3], which achieved good results on two relation extraction datasets with more overlapping ternaries, NYT and WebNLG. The model contains the head entity decoder and the relation-specific tail entity annotator.

#### Head entity decoder

The sentence to be extracted is first encoded as an embedding vector through the BERT (Bidirectional Encoder Representation from Transformers) layer [34], then decoded by a head entity decoder that decodes all possible head entities. The structure of the head entity decoder is two binary categorical annotators. The probability of the annotator at each position in the sentence is calculated as follows:

$$\begin{array}{c} p_{i}^{start\_h}=\sigma \left(W_{start}X_{i}+b_{start}\right) \end{array}$$ (1)

$$\begin{array}{c} p_{i}^{end\_h}=\sigma \left(W_{end}X_{i}+b_{end}\right) \end{array}$$ (2)

where *X*_*i*_ = *h*_*N*_[*i*] denotes the embedding representation of the *i*-th position in the sentence vector, *W*_*start*_ and *W*_*end*_ denote the trainable weights, *b*_*start*_ and *b*_*end*_ are the offsets, and *σ* is the Sigmoid activation function. $p_{i}^{start\_h}$ and $p_{i}^{end\_h}$ denote the probability which the annotator computes that the *i*-th position in the sentence is the start and end position of the head entity. If the probability is more significant than a given threshold, the position is considered the first or last position of the head entity and its label is set to 1, otherwise, it is set to 0. The head entity decoder optimizes the following likelihood function to identify the head entity position for a given sentence *X*:

$$\begin{array}{c} p_{\theta }\left(s|X\right)=\underset{t\in \{start\_h,end\_h\}}{\Pi }\overset{L}{\underset{i=1}{\Pi }}{\left(p_{i}^{t}\right)}^{I\{y_{i}^{t}=1\}}{\left(1-p_{i}^{t}\right)}^{I\{y_{i}^{t}=0\}} \end{array}$$ (3)

where *L* is the length of the sentence; $y_{i}^{start\_h}$ and $y_{i}^{end\_h}$ denote the label that the *i*-th position is the start and end position of the head entity; *I*(*z*) = 1 if *z* is true, otherwise *I*(*z*) = 0; parameter *θ* = {*W*_*start*_, *W*_*end*_, *b*_*start*_, *b*_*end*_}. When there are multiple head entities in a sentence, the model looks for its closest end tag as an entity for each start tag.

#### Relation-specific tail entity annotator

Each relation-specific tail entity annotator will annotate the corresponding tail entities for all the decoded head entities. Since it is necessary to decode the tail entity corresponding to the head entity, the input vector of the tail entity annotator needs to integrate the information of the head entity, and its probability formula for each position is as follows:

$$\begin{array}{c} p_{i}^{start\_t}=\sigma \left(W_{start}^{r}\left(X_{i}+V_{sub}^{k}\right)+b_{start}^{r}\right) \end{array}$$ (4)

$$\begin{array}{c} p_{i}^{end\_t}=\sigma \left(W_{end}^{r}\left(X_{i}+V_{sub}^{k}\right)+b_{end}^{r}\right) \end{array}$$ (5)

where $p_{i}^{start\_t}$ and $p_{i}^{end\_t}$ denote the probability which the annotator computes that the *i*-th position in the sentence is the start and end position of the tail entity, and $V_{sub}^{k}$ denotes the mean of the vector for each position of the *k*-th head entity. The relation-specific tail entity annotator identifies the tail entity positions after a given sentence *X* and head entity *h* by optimizing the following likelihood function:

$$\begin{array}{c} p_{\Phi_{r}}\left(t|s,X\right)=\underset{t\in \{start\_t,end\_t\}}{\Pi }\overset{L}{\underset{i=1}{\Pi }}{\left(p_{i}^{t}\right)}^{I\{y_{i}^{t}=1\}}{\left(1-p_{i}^{t}\right)}^{I\{y_{i}^{t}=0\}} \end{array}$$ (6)

where $y_{i}^{start\_t}$ and $y_{i}^{end\_t}$ are the labels of the start and end positions of the tail entity at the *i*-th position in the sentence *X*. For all positions in the “empty” tail entity $t_{⌀}$, there is $y_{i}^{start\_t}=y_{i}^{end\_t}=0$. And parameter $\Phi_{r}=\left\{W_{start}^{r},W_{end}^{r},b_{start}^{r},b_{end}^{r}\right\}$.

In the post-order extraction step, the relation is also determined by the output of the tail entity annotator. In this case, the high-level module can recognize relations and tail entities related to the head entities detected in the low-level module. However, the original structure of the CasRel model suffers from two problems: first, the head entity decoder is the bottleneck of the model’s relation extraction performance, pending the use of a better-performing head entity decoder; and second, its tail entity decoder suffers from sample imbalance, which affects the performance of its tail entity decoder.

### 3.2 CasRelBLCF model

Aiming at the above-mentioned problems, in this paper, we propose a joint model relation extraction method based on entity mapping (CasRelBLCF), which is improved based on the CasRel extraction model. Firstly, a joint Bi-LSTM and CRF head entity decoder is proposed, focusing on improving the performance of the head entity in extracting relational triples from unstructured text. Then, the Focal Loss function is used to alleviate the sample imbalance problem of the model in tail entity decoding. Moreover, given that the noisy data contained in the dataset, especially obtained by distant supervision, affects the training of the model and reduces the performance of the model in extracting the triples, we design a reinforcement learning-based distant supervised noise reduction method to mitigate the impact of noise in the distant supervised dataset on the model, to improve the accuracy of the relation extraction effectively.

**Fig 1:**
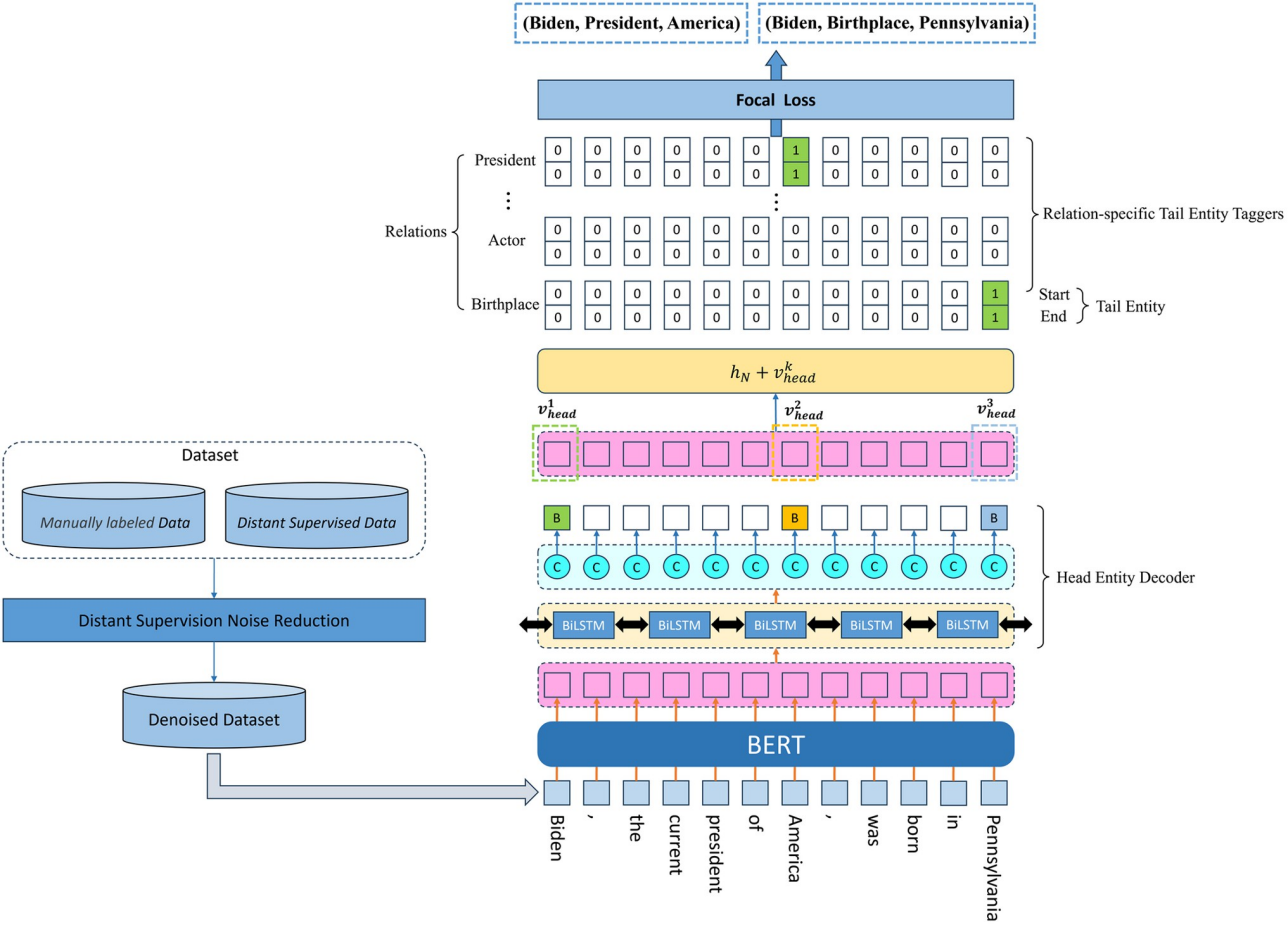
Structural diagram of the CasRelBLCF model and the pipeline in training stage.

Fig 1 displays the architecture of CasRelBLCF model and the pipeline in training stage. The model starts with a BERT encoder, and sentences to be extracted are fed to it for obtaining their embeddings, which are then input into a Bi-LSTM+CRF decoder to identify all the head entities, where we use BIO annotation method. After that, the feature of head entity and sentence are summed and fed into each relation-specific tail entity tagger to annotate the tail entity for each relation of the head entity. In the training stage, a data preprocessing is added, in which the reinforcement learning-based distant supervised noise reduction method we propose is used for distant supervised training set. Then, the denoised dataset is input into the CasRelBLCF model and corresponding Focal Loss is computed, which is used to update the gradient of the whole model. Additionally, it has been confirmed through numerous experiments that in order to address the issue of imbalance between the loss functions of the head entity decoder and the tail entity decoder, the model needs to use sentence embeddings that are encoded from two independent BERT encoders for the head entity decoder and the tail entity decoder, otherwise, the model will not converge.

### 3.3 Head entity decoder

The CasRel model is designed to address the issue of overlapping triples in relation extraction. In this problem, the head entity decoder does not consider sentence continuity information, and the tail entity decoder faces the problem of sample imbalance. To overcome these limitations and enhance the performance of the entity decoder in relation extraction, the CasRel model aims to improve the head entity extraction performance. In this paper, we design a head entity decoder based on entity mapping, which pays more attention to sentence continuity. Firstly, a bi-directional long short-term memory neural network Bi-LSTM is used for further feature extraction of the BERT-encoded sentence vectors, and then a conditional random field CRF is introduced to decode the head entities in the sentences. This modeling approach has obvious advantages over the BERT model. One is that Bi-LSTM has a specially designed gate mechanism, which can effectively alleviate the gradient vanishing problem and achieve long-distance dependency. CRF is a conditional probability distribution model for solving another set of output sequences given input sequence conditions. CRF is widely used in sequence labeling problems [35] because the conditional random field will compute both the before and after state information before judging the current position label, compared with directly using the BERT encoded sentence for decoding, the sentence vector encoded using Bi-LSTM has more long-distance dependency information of the words before and after. While using CRF for decoding, the continuity of the sentence will be more sufficiently considered, reducing the probability of illegal labels.

### 3.4 Loss function

**Fig 2:**
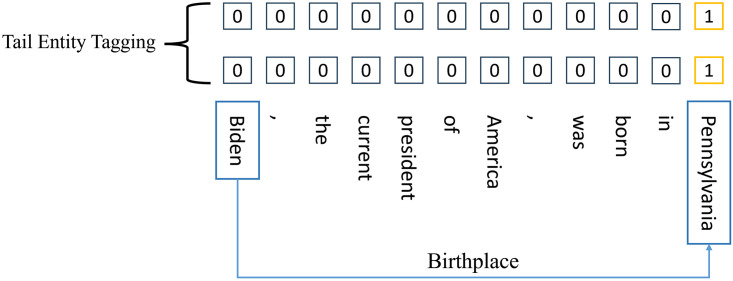
An illustration of sample imbalance problem in the tail entity tagging.

CasRel model use two binary categorical annotators to tag the start and end position of tail entities, where there is a severe sample imbalance problem which has a significant adverse impact on the training effectiveness of the model. An illustration is shown in Fig 2. To extract the relational triple <*Biden*, *Birthpalce*, *Pennsylvania*> in the sentence “Biden, the current president of America, was born in Pennsylvania”, only the start and end position of tail entity “Pennsylvania” will be tagged with “1”, and all of other position in the tail entity labeling sequence will be tagged with “0”, where the proportion of label “1” is only 8.3%. We also investigate the situation of sample imbalance in the four commonly employed public datasets in the field of relation extraction: webNLG, NYT, NYT11-HRL and DuIE2.0 (the detail of these datasets will be introduced in Section 4 Experiments), as shown in Table 1. This imbalance will make the tail entity tagger more inclined to annotate label “0” and learn how to correctly annotate label “1” less easily.

**Table 1:** The proportion of label “1” in tail entity labeling sequence of webNLG, NYT, NYT11-HRL and DuIE2.0.

Dataset	Proportion of label “1” (%)
webNLG	10.37
NYT	4.76
NYT11-HRL	3.41
DuIE2.0	3.41

In order to solve the sample imbalance problem, this paper adopts the Focal Loss function [36] as the loss function of the tail entity annotator. Focal Loss contains two attributes: **(1)** when there are many samples in a specific instance, and the model learns better, it is close to 1, close to 0, and the loss value will be reduced because of this factor; on the contrary, when there are fewer samples in a specific instance, and the model learns worse, it is close to 1, close to 0, and the loss value will decrease because of this factor; on the contrary, when there are fewer samples in an instance, and the model learns When the model learns poorly, it is close to 1, and the loss value is hardly affected by the factor. **(2)** The parameter can regulate the degree of Focal Loss bias toward difficult-to-categorize samples. When the more significant, the degree of the model tends to difficult-to-categorize samples will increase. According to Eq (7), we assign the parameters. Then, according to Eq (8), for p closer to the position of 0.5, its final Focal Loss value will be higher. Can effectively reduce the Loss value of the negative samples (simple samples). The greater the probability of the simple samples, the stronger the effect. The greater the probability of the simple samples, the more the loss of the simple samples can be reduced to a lower level.

$$\begin{array}{c} FL\left(P_{t}\right)=-\alpha {\left(1-p_{t}\right)}^{r}\text{log}\left(p_{t}\right) \end{array}$$ (7)

$$\begin{array}{c} P_{t}=\{\begin{array}{cc} p & if\;y=1\\ 1-p & otherwise \end{array}bu \end{array}$$ (8)

In the original CasRel model, to predict a position in the sentence “0” is a simple sample. The model for the prediction of “0” will be powerful, so the predicted probability value tends to be far away from the position of 0.5. This prediction of simple samples This ability to predict simple samples is different from what the model should focus on training. On the contrary, the tail entity that needs to be extracted, whose label is “1”, this kind of entity accounts for a deficient proportion of the whole sentence. Hence, its prediction difficulty is more serious, and the predicted probability value will be close to 0.5. Therefore, for the sample with the label “1”, which is more difficult to predict correctly, the model can be trained using the “1” model. Therefore, for samples like label “1”, which are more difficult to predict correctly, the adjustment of the Focal Loss function can significantly improve its loss, which allows the model to pay more attention to learning how to predict this kind of indistinguishable samples. Focal Loss function reduces the model’s attention to the learning of the label “0” and improves the model’s attention to the label “1”. The Focal Loss function reduces the model’s focus on the label “0”. It increases its focus on label “1”, which is the label of the tail entity that we need to extract, which also alleviates the sample imbalance problem mentioned earlier. With the mediation of the Focal Loss function, the model will enhance its ability to predict the tail entities. Eventually, the extraction precision and recall of the model will be improved.

### 3.5 Distant supervised noise reduction method

In order to eliminate the effect of noise on the accuracy of relation extraction, Qin et al. [37] used a deep reinforcement learning strategy for distant supervised datasets to filter false-positive noise, which automatically identifies noise for each relation type without any supervisory information. This deep reinforcement learning-based noise reduction can be used as a pre-processing step to first reduce the noise in the dataset without making changes to the CasRelBLCF relation extraction model itself. But because a sentence can react to a particular relation does not mean that all triple instances of that sentence can react to that relation, the granularity is too large for sentences with many triples in this noise reduction method. Inspired by this, This paper proposes a novel noise reduction method based on reinforcement learning as a pre-processing plug-in for the CasRelBLCF model to reduce impact of noise in distant supervised data.

Based on the work of Qin et al. in this paper, we change the status of the agent to the combination of the information of the head entity and that of the sentence encoding. As shown in Eq (9), where *h*_*ins*_ is the instance vector, *h*_*hp*_ is the head entity position, and *h*_*a*_ is the sentence embedding vector.

$$\begin{array}{c} h_{ins}=h_{hp}*h_{a}+h_{a} \end{array}$$ (9)

**Fig 3:**
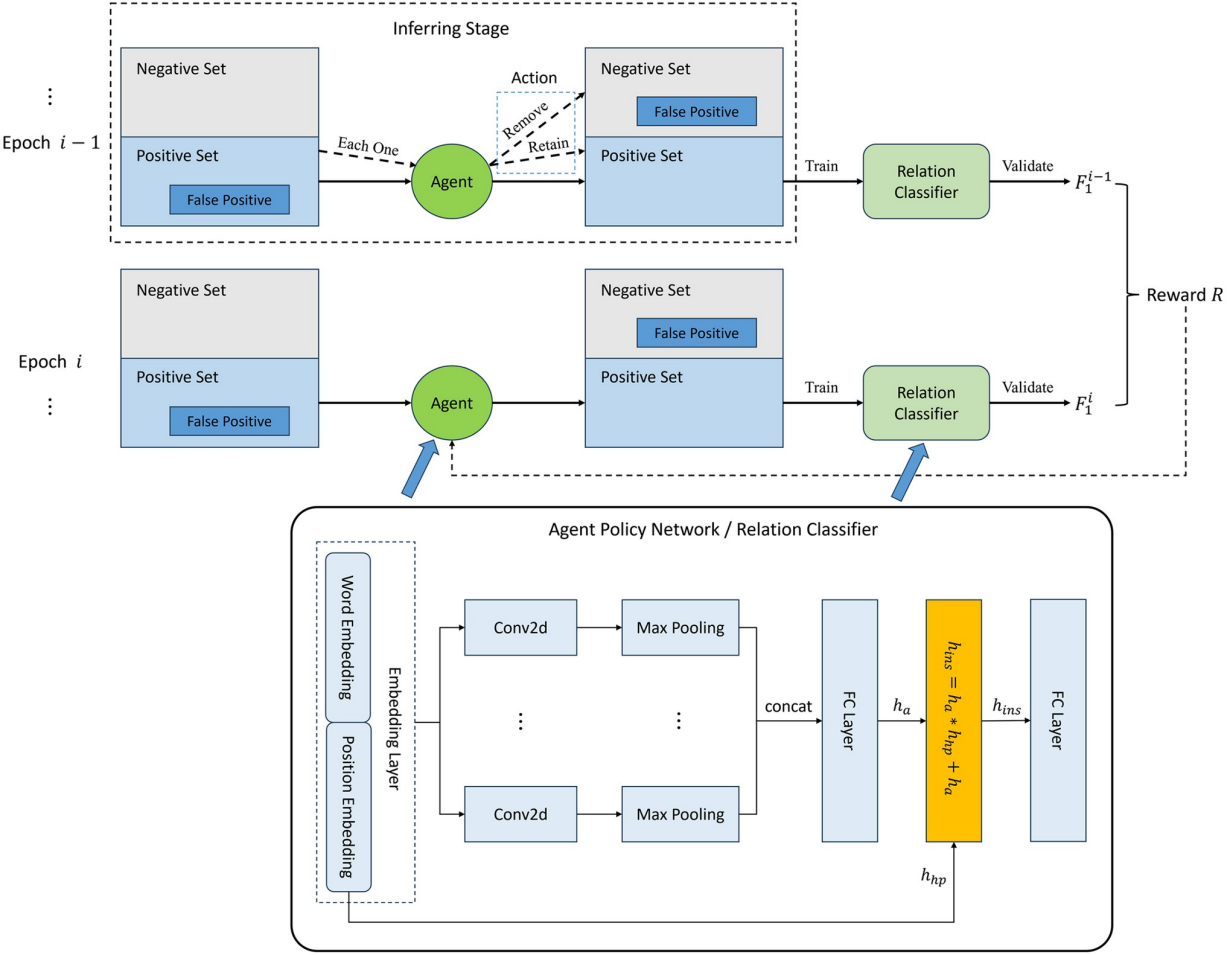
Deep reinforcement learning noise reduction process.

The overall framework of our reinforcement learning-based distant supervised noise reduction method is shown in Fig 3, where each relation type has a agent for denoising. For a relation type *Rel*, all the sentences containing triples belonging to *Rel* are regarded as positive set, and the other is regarded as negative set, but due to the defect of distant supervision, there are usually some false-positive noise in the positive set, which refers to the samples in which an entity pair does not reflect relation *Rel* but is incorrectly labeled. In the inferring stage, the agent of *Rel* is used to determine whether each sentence in positive set is a false-positive sample, corresponding to two action: remove and retain, respectively, and positive set removed false-positive samples is the denoised dataset for relation type *Rel*.

To train the agent, in each epoch, we use the dataset redistributed by agent to train a extra relation classifier and validate it for obtaining *F*_1_ score, and the reward *R*_*i*_ of epoch *i* can be calculate by the *F*_1_ scores of epoch *i* − 1 and epoch *i*, which is the same as Qin et al., as shown in Eq (10):

$$\begin{array}{c} R_{i}=\alpha \left(F_{1}^{i}-F_{1}^{i-1}\right) \end{array}$$ (10)

where *α* is a hyperparameter. The policy network of agent and the relation classifier both use a simple CNN network with a feature fusion layer employing the fusion strategy in Eq (9). Moreover, in order to Accelerate the training speed of reinforcement learning, we use the dataset before denoising divided into positive and negative sets to pretrain each agent, similar to the training process of the extra relation classifier.

## 4 Experiments

In this section, the improved method proposed in the previous section is experimented and analyzed. The experiments use publicly available relation extraction datasets to train the CasRelBLCF model. The improved performance is evaluated using standard metrics for relation extraction tasks, and finally, the experiment results are analyzed and summarized.

### 4.1 Datasets and setup

The WebNLG dataset was created for the Natural Language Generation (NLG) task and adapted to the Relational Triples Extraction dataset by Zeng et al. [19] in 2018. It contains 246 predefined relation types. The training set of WebNLG contains 5019 sentences, the validation set contains 500 sentences, and the test set contains 703 sentences.

The NYT dataset was first released by Riedel et al. [38], but we use the filtered version proposed by Zeng et al. [19]. Its training set contains 56,195 sentences, and both validation and test sets contain 5,000 sentences respectively. Moreover, all of them are generated by distant supervision techniques.

The NYT11-HRL dataset was compiled by Takanobu et al. in 2019. Here are two versions of the NYT dataset: the original version of the training set and test set was generated by distant supervision by Riedel et al. In another smaller version with fewer relation types, the training set was generated by distant supervision, but the test set was munually labeled by Hoffmann et al. in 2011. Here, we denote the original and smaller versions as NYT10 and NYT11, respectively. To fairly compare these models, Takanobu et al. [17] applied uniform preprocessing to NYT10 and NYT11 and made a complete comparison with previous work using the same dataset. Here, we denote the two preprocessed versions as NYT10-HRL and NYT11-HRL, respectively. Statistics for the three datasets are shown in Table 2.

**Table 2:** Statistics of WebNLG, NYT and NYT11-HRL.

Dataset	#Train	#Validation	#Test
webNLG	5019	500	703
NYT	56195	5000	5000
NYT11-HRL	60894	0	369

The DuIE2.0 dataset is currently the most significant Chinese relation extraction dataset. The public training set has more than 170,000 sentences and 310,000 relational triples, the validation set has 20,674 sentences and 37,825 relation triples, and the test set is not public. Most of the sentences in the dataset come from Baidu’s information texts on Internet-related products, such as Baidu Postings and Baidu Encyclopedia. The instances in the dataset are automatically labeled and generated by distant supervision. Because the test set of this dataset is not publicly available, in this paper, to compare the model improvement performance, 10% of the training set is randomly selected as the validation set, and the validation set of the original dataset is used as the test set. The information of the original DuIE2.0 dataset is shown in Table 3.

**Table 3:** Statistics of DuIE2.0 dataset.

Dataset	DuIE2.0		
	#Sentences	#Triples	#Relations
Train	171293	310709	48
Validation	20674	37825	48

In this paper, we retain the original division of training, validation and test sets of all the above-mentioned datasets. And the main hardware and software environments we use are shown in Tables 4 and 5.

**Table 4:** The main environment of hardware.

Hardware	Model/Capacity
OS	Linux
Memory	64GB
Hard Disk	2T
GPU	RTX3090

**Table 5:** The main environment of software.

Software	Version
Python	3.7
Torch	1.8.0+cu111
Transformers	4.3.3
FastNLP	0.6.0
Tqdm	4.59.0
Numpy	1.20.1

### 4.2 Relation extraction task and metrics

The relation extraction task refers to extracting all correct triples which can be represented by <*Head*_*Entity*, *Relation*, *Tail*_*Entity*> when extracted from a sentence. It is worth noting that there are two different evaluation criteria in previous triple extraction tasks: **(1)** a widely used one is partial matching, which means that the extracted relational triple <*Head*_*Entity*, *Relation*, *Tail*_*Entity*> is considered correct only if both the relation and head positions of the head and tail entities are correct; and **(2)** a more stringent but less popular one is that employed by Dai et al. [39] where the extracted relational triple <*Head*_*Entity*, *Relation*, *Tail*_*Entity*> is considered correct only if the relation between the head and tail entities and both the head and tail positions of entities are correct. In this paper, we use partial matching for metrics following the evaluation criteria of the original CasRel model. Precision, Recall, and F1-Score are used as the evaluation metrics, as shown in Table 6.

**Table 6:** Evaluation metrics.

Metrics	Description
Precision	Number of correctly extracted triples/Number of extracted triples
Recall	Number of correctly extracted triples/Number of all correct triples
F1	$\text{2}\times \frac{\text{Precision}\times \text{Recall}}{\text{Precision}+\text{Recall}}$

### 4.3 Comparison with previous work

We select some representative baseline approaches. **NovelTagging** [40] proposes to convert the joint extraction task into a tagging problem. The model consists of a bidirectional long-time memory layer (Bi-LSTM) to encode the inputs and an LSTM-based decoding layer with bias loss. The bias loss enhances the relevance of entity labels. **CopyRE** [19] proposes an end-to-end learning model based on a copying mechanism that jointly extracts relevant facts from sentences of these classes. The model proposes for the first time to consider the problem of relational triple state overlap through the copy mechanism. **GraphRel** [41] proposes an end-to-end relations extraction model using Graph Convolutional Networks (GCNs) to learn named entities and relations jointly. **SPointer** [42] proposes an end-to-end model with a two-pointer module that jointly extracts entire entities and relations, where multi-layer convolution and self-attention mechanisms are used as encoder to learn the correlation between long-distance entities. **CopyRRL** [43] proposes a sequence-to-sequence model based on reinforcement learning and discusses the problem of multiple triples extraction ordering. **Relation Aware** [44] proposes an improved model for the joint extraction of entities and relations by GCNs. The joint entity and relation extraction is divided into two subtasks, including entity span detection and entity-relation type recognition. A new relation-aware attention mechanism is proposed for obtaining relation features. **CasRel** [3] proposes a new cascading binary tagging framework that models relations as functions mapping topics to objects, which can simultaneously extract multiple relational triples from sentences and effectively solve the problem of overlapping triples. **PMEI** [5] devised a progressive multi-task learning model with explicit interaction, utilized early-predicted interactions to refine task-specific representations. **SGNet** [7] proposes a joint extraction model based on Soft Pruning and GlobalPointer. By leveraging BERT pre-trained models to obtain text word vectors enriched with contextual information. **BCT** [20] composes an end-to-end BCT framework to extract the overlapping entities and relations jointly. Unlike previous sequential frameworks, it utilize an efficient binary cross-matching method for constructing entities that participate in multiple triples. The reported results of the above baseline are directly copied from the original papers. Ours CasRelBLCF model is compared with the baseline approaches from three perspectives, namely Precision, Recall, and F1-Score, and it is verified that our approach works best in carrying out the relation extraction task through experiments on two publicly available datasets, NYT and WebNLG. The experiment results are detailed in Table 7.

**Table 7:** Experiment results of CasRelBLCF and all the baselines on NYT and WebNLG.

Models	NYT			WebNLG		
	Precision	Recall	F1	Precision	Recall	F1
NovelTagging	62.4	31.7	42.0	52.5	19.3	28.3
CopyRE_OneDecoder_	59.4	53.1	56.0	32.2	28.9	30.5
CopyRE_MultiDecoder_	61.0	56.6	58.7	37.7	36.4	37.1
GraphRel_1p_	62.9	57.3	60.0	42.3	39.2	40.7
GraphRel_2p_	63.9	60.0	61.9	44.7	41.1	42.9
SPointer	72.8	69.0	70.9	38.7	37.5	38.1
CopyRRL	77.9	67.2	72.1	63.3	59.9	61.6
Relation-Aware	83.2	64.7	72.8	66.4	62.7	64.5
CasRel	89.7	89.5	89.6	93.4	90.1	91.8
PMEI	90.5	89.8	90.1	91.0	**92.9**	92.0
BCT_BERT_	89.8	88.5	88.9	90.6	92.0	91.3
SGNet	91.2	**91.4**	**91.3**	91.8	91.9	91.9
**CasRelBLCF(Ours)**	**91.6** ↑+2.1%	89.2 ↓-0.3%	90.4 ↑+0.9%	**93.5** ↑+0.1%	91.9 ↑+2.0%	**92.7** ↑+1.0%

In the baseline models, NovelTagging, CopyRE, GraphRel, and SPointer are end-to-end models using joint extraction to learn named entities and relations using machine learning methods such as bi-directional long-time memory layers (Bi-LSTM), GCN, multi-layer convolution and self-attention mechanism. But the precision of these models is relatively poor, where the highest can only reach 72.8% on the NYT dataset and 38.7% on the WebNLG dataset. CopyRRL and Relation-Aware extract relations from the perspective of reinforcement learning and attention mechanism respectively, which improves the performance of model. The highest precision of Relation-Aware on the NYT dataset reaches 83.2%, and improvement is more significantly on WebNLG dataset, with a 72% improvement for precision, 40% for recall and 41% for F1-score compared to SPointer.

In 2020, the newly proposed CasRel model addressed the challenge of overlapping triple problem effectively. It achieved an precision of 89.7% on the NYT dataset and demonstrated a notable improvement with a 93.4% precision on the WebNLG dataset. In 2021, The PMEI model, introduced following a progressive multi-task learning approach with explicit interaction, refines task-specific representations leveraging early predictions. On the NYT dataset, the PMEI model achieves an precision of 90.5%, and meanwhile, attains an average precision, recall and F1-score of 92% on the WebNLG dataset, highlighting the efficacy of multi-task learning in joint entity and relation extraction tasks. In 2022, the *BCT*_*BERT*_ model was presented as an end-to-end BCT framework capable of jointly extracting overlapping entities and triplets. Compared to the CasRel model, it demonstrates slight improvements in certain metrics on the WebNLG dataset. Also in 2022, the SGNet model, a novel joint extraction model, was proposed. It relies on soft pruning and a global pointer mechanism, utilizing BERT pre-trained models to acquire text word vectors enriched with contextual information. SGNet excels in overlapping triple extraction, achieving an average precision, recall and F1-score of 91.3% on the NYT dataset. On the WebNLG dataset, although its precision is slightly lower than the CasRel model by 1.7%, it still achieves an impressive average precision, recall and F1-score of 91.9%. The extraction effect of the above methods in the two datasets has been greatly improved, but there are still problems such as unbalanced labeled samples and insufficient consideration of sentence continuity by its head entity decoder, and the precision of the relation extraction still needs to be further improved.

The CasRelBLCF model is improved based on the CasRel extraction model, which mainly contains three aspects: one is the head entity decoder with joint Bi-LSTM and CRF, the second is the use of the Focal Loss function, and the third is the distant supervised noise reduction method based on reinforcement learning. When our model carries out the relation extraction task in two benchmark datasets, it achieves effective improvement in all three aspects: precision, recall, and F1-score. Compared with CasRel, our CasRelBLCF model improves precision by 2.1%, F1-score by 0.9% on the NYT dataset, although recall is slightly lower by 0.3%. On the WebNLG dataset, precision improves by 0.1%, F1-score improves by 1%, and recall improves by 2%. In addition, when compared to the latest SGNet model, our model exhibits a 0.4% improvement in precision on the NYT dataset and a 2% improvement in precision along with a 0.9% increase in F1-score on the WebNLG dataset, where the recall performance also remains comparable. On the NYT dataset, our model outperforms the partial baseline, and compared to the latest model, its average performance is slightly lower by 0.1%. However, on the WebNLG dataset, where other baselines show mediocre performance, our model achieves the highest average metrics at 92.7%.

**Fig 4:**
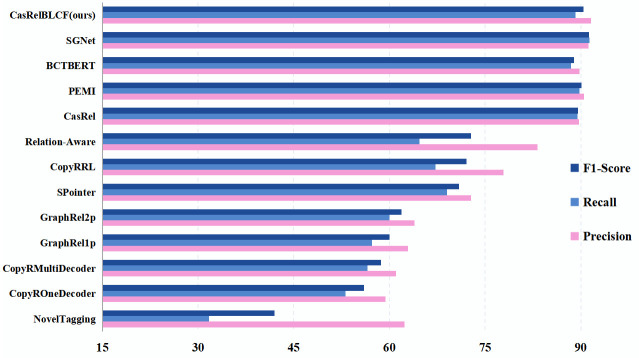
Visualized experiment results on NYT.

**Fig 5:**
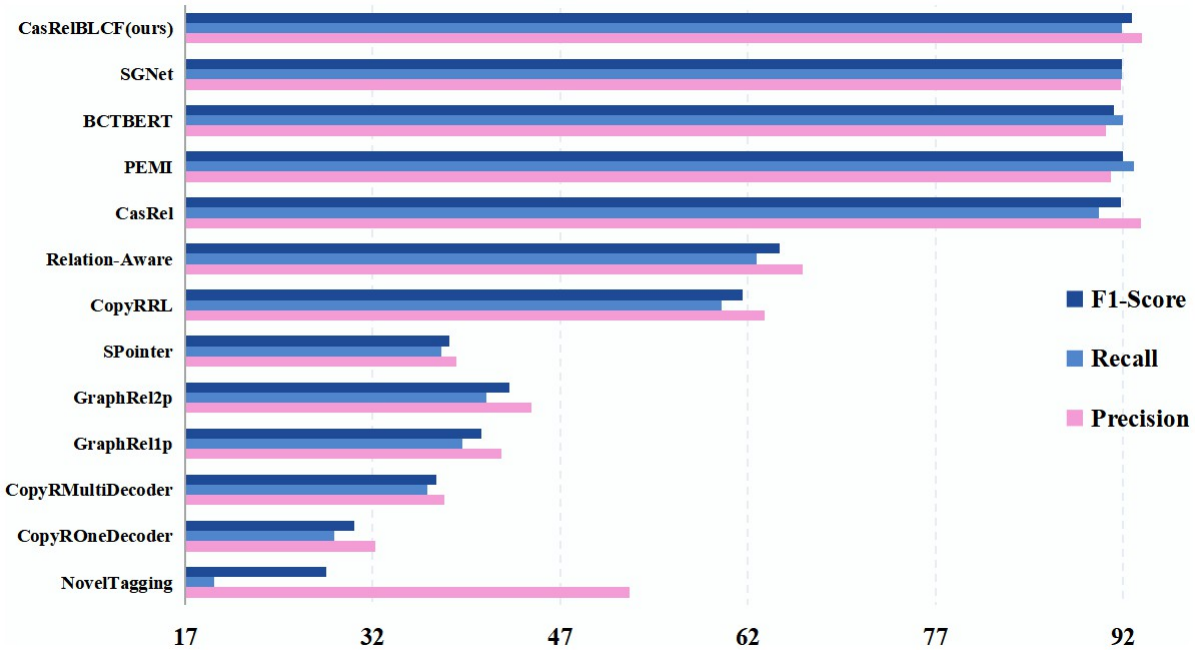
Visualized experiment results on WebNLG.

The results indicate that our method can address the impact of sample imbalance effectively and pay better attention to the continuity of sentences in head entity decoding on the ternary relation extraction ability and further enhance the model’s performance in extracting relational triples from unstructured text. More intuitively, the visualized experiment results on NYT and WebNLG are shown in Figs 4 and 5.

### 4.4 Ablation experiment

To assess the effectiveness of the proposed model, this paper conducts ablation experiments on three benchmark datasets. The evaluation is based on three metrics commonly used in classification: Precision, Recall and F1-Score. In the ablation experiment, we use four schemes to validate the effectiveness of our model.

#### 4.4.1 Validation of head entity decoder

To verify the excellent performance of the proposed model in the relation extraction task, our model is compared with the CasRel model on three English datasets. The main parameters of the Bi-LSTM+CRF head entity decoder are set as follows: the hidden size is 128, the number of layers in Bi-LSTM is 3, the dropout is 0.5, the number of label types in CRF decoder is 3, and its predicted labels are set as: ‘O’, ‘B-HEAD’, ‘I-HEAD’, where ‘B-HEAD’ indicates the start position of an head entity, ‘I-HEAD’ indicates other position in an head entity except the start position, and ‘O’ indicates the position not belonging to any head entity. The experiment is conducted on three English datasets, NYT, WebNLG and NYT11-HRL, using the above parameters, and the results are shown in Table 8, in which CasRelBLCF model without using Focal Loss and the improved distant supervised noise reduction method is represented by CasRelBLC.

**Table 8:** Experiment results of head entity decoder validation.

Models	NYT			WebNLG			NYT11-HRL		
	Precision	Recall	F1	Precision	Recall	F1	Precision	Recall	F1
CasRel	**89.7**	89.5	89.6	**93.4**	90.1	91.8	50.1	**58.4**	53.9
**CasRelBLC**	89.3 ↓-0.4%	**89.9** ↑+0.4%	**89.6** -	91.6 ↓-2.0%	**92.2** ↑+2.3%	**91.9** ↑+0.1%	**52.0** ↑+3.8%	57.6 ↓-1.3%	**54.6** ↑+1.2%

The experiment shows that the improved head entity decoder brings some improvement on the NYT11-HRL dataset, with the F1-score increasing from the original 0.539 to 0.546. However, there is little promotion on the WebNLG and NYT datasets, which may be attributed to that previous relation extraction models, such as the one proposed by Zeng et al. [19], can only recognize the information about the first position of the entity, therefore the WebNLG and NYT datasets they filter only annotate the head position of the entity. The coarse-grained labels lead to a significant reduction in the difficulty of entity recognition, therefore, the head entity recognition performance has reached the upper limit, and it is challenging to continue to improve. However, the NYT11-HRL dataset labels the whole entity’s position entirely, so our model improves the F1-score on the NYT11-HRL dataset by 0.7 percentage points, which verifies that Bi-LSTM+CRF as the head entity decoder of the CasRel model can significantly improve its comprehensive performance of relation extraction.

#### 4.4.2 Validation of Focal Loss function

We use Focal Loss as the loss function of the tail entity decoder to alleviate the sample imbalance problem of the model in tail entity decoding, with the experimental parameters *α* = 0.25, *γ* = 2, where *α* is the category weights used to adjust the proportion of the loss function between different categories and *γ* is the difficulty weights, which adjusts the loss value of the more challenging samples higher.

The CasRelBLCF model was trained using the above parameters and the improved head entity decoder. The F1-score increases on all three datasets, NYT, WebNLG and NYT11-HRL, after improving the head entity decoder and loss function on the CasRel model improves. The F1-score on the WebNLG dataset is improved from 0.918 to 0.927, and those on the NYT and NYT11-HRL datasets are improved from 0.896 to 0.904 and from 0.539 to 0.550, respectively. The experiment results show that using the Focal Loss function can significantly alleviate the sample imbalance problem in the tail entity decoder of the CasRelBLC model and effectively improve the performance of relation extraction. The results are shown in Table 9, where CasRelBLCF indicates the our model without the improved distant supervised noise reduction method.

**Table 9:** Experiment results of Focal Loss function validation.

Models	NYT			WebNLG			NYT11-HRL		
	Precision	Recall	F1	Precision	Recall	F1	Precision	Recall	F1
CasRel	89.7	**89.5**	89.6	93.4	90.1	91.8	50.1	**58.4**	53.9
**CasRelBLCF**	**91.6** ↑+2.1%	89.2 ↓-0.3%	**90.4** ↑+0.9%	**93.5** ↑+0.1%	**91.9** ↑+2.0%	**92.7** ↑+1.0%	**54.5** ↑+8.8%	55.4 ↓-5.1%	**55.0** ↑+2.0%

#### 4.4.3 Validation of distant supervised noise reduction method

We validated the effectiveness of distant supervised noise reduction using the NYT11-HRL dataset. The training parameters of the CasRelBLCF model and the deep reinforcement learning noise reduction filter are as follows: the maximum sentence length for reinforcement learning training is 120, and sentences exceeding this length are truncated; the maximum distance of the position features is 100; the dimension of the position matrix is 100*2+2; hidden size of CNN policy network is 100; hidden size of CNN relation classifier is 100; the dimension of word embedding is 50; the dimension of position embedding is 5; the batch size of reinforcement learning is 160; learning rate of reinforcement learning is 2e-5; the dropout is 0.5; reward scale for reinforcement learning is 100. After using the above parameters for the training of reinforcement learning and noise filtering on the NYT11-HRL dataset, the results are shown in Table 10.

**Table 10:** Validation of the effectiveness of distant supervised noise reduction method.

NYT11-HRL	#Sentences	Labeling method
Original training set	60894	Distant Supervision
Filtered training set	57549	Distant Supervision + RL Filtering
Test set	370	Manual labeling

**Fig 6:**
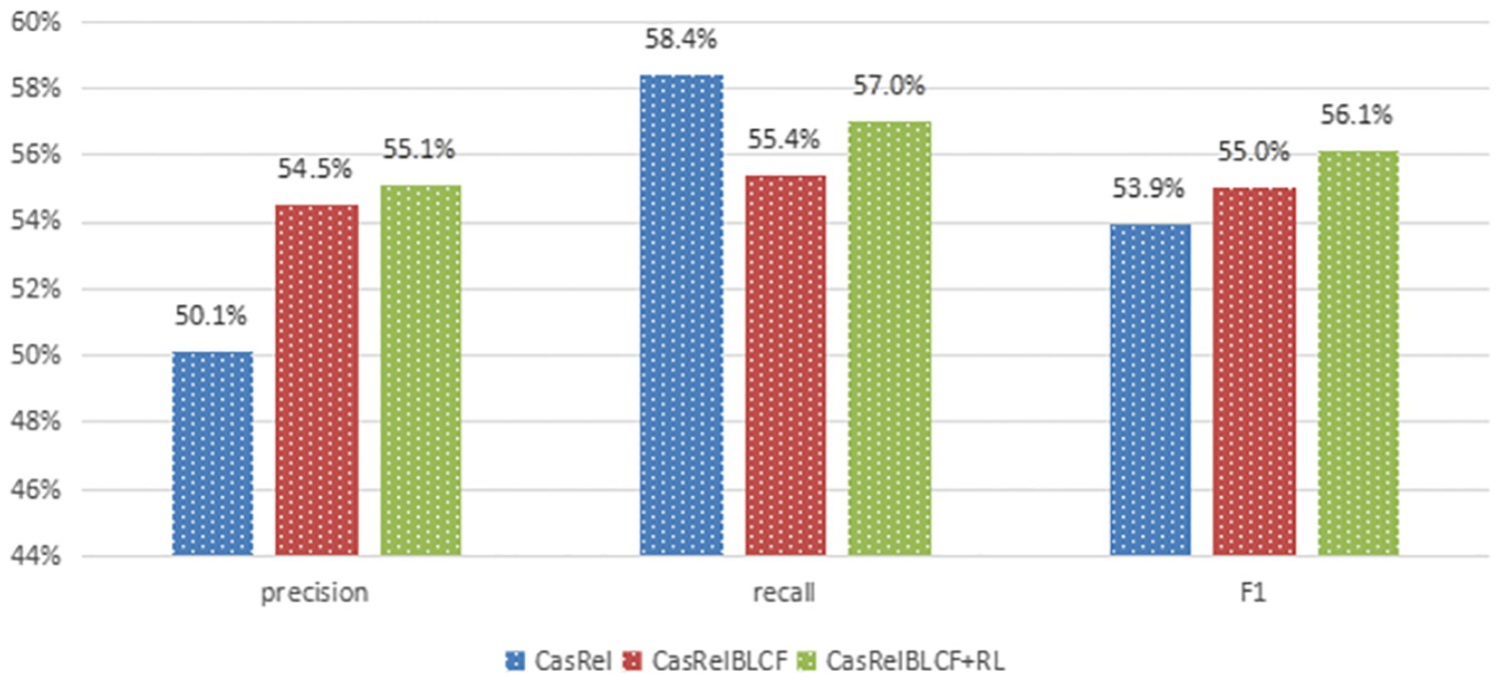
Visualized experiment results on NYT11-HRL dataset.

The above results show that after training and filtering with deep reinforcement learning, the training set of the NYT11-HRL dataset was filtered from the initial 60894 sentences to 57549 sentences, and 3345 sentences were filtered out by the reinforcement learning agent, recognizing them as sentences containing false positive noise. We use the filtered NYT11-HRL dataset to retrain the CasRelBLCF model and test it. The results show that after using reinforcement learning for noise reduction on the dataset NYT11-HRL, the model’s training effect is further improved, and the F1-score on the test set is improved from 0.550 to 0.561. It indicates that the noise reduction module with deep reinforcement learning can effectively reduce the noise in the distant supervised dataset and improve the training effect of the CasRelBLCF model on the distant supervised dataset. The details are shown in Table 11 and Fig 6.

**Table 11:** Experiment results on NYT11-HRL dataset.

Models	NYT11-HRL		
	Precision	Recall	F1
CasRel	50.1	58.4	53.9
CasRelBLCF	54.5	55.4	55.0
**CasRelBLCF+RL**	**55.1** ↑+1.0%	**57.0** ↑+3.0%	**56.1** ↑+2.0%

#### 4.4.4 Validation on Chinese dataset

To validate the effectiveness of CasRelBLCF model on Chinese dataset, we train the original CasRel model and the improved CasRelBLCF model on the DuIE2.0 dataset and the test results are shown in Table 12. The F1-score of our model is 2% higher than that of the original CasRel model, which indicates the more comprehensive performance of our model in relation extraction task on Chinese dataset.

**Table 12:** Experiment results on Chinese dataset.

Models	DuIE2.0		
	Precision	Recall	F1
CasRel	**75.5**	65.2	69.9
**CasRelBLCF**	74.0 ↓-2.0%	**68.6** ↑+5.0%	**71.2** ↑+2.0%

## 5 Conclusion

This paper proposes a novel approach called CasRelBLCF to address challenging problems in relation extraction, specifically triplet overlap, insufficient consideration of sentence continuity by the head entity decoder, sample imbalance and noise in distant supervision. We proposed the joint Bi-LSTM and CRF decoder, which aims to enhance the extraction of head entities from unstructured text, thereby improving the performance of relational triple extraction. The Focal Loss function is used as a solution to address the issue of sample imbalance in tail entity decoding. Because the presence of noisy data in the dataset would negatively impact the model training and hamper its performance in extracting the triples, a noise reduction method based on reinforcement learning is designed to mitigate the impact of noise in the distant supervised dataset, thereby enhance the accuracy of relation extraction.

The effectiveness of the CasRelBLCF model is validated in terms of the head entity decoder, sample imbalance, and distant supervised noise reduction. The experiment results on three public English datasets and the DuIE2.0 Chinese dataset show that the F1-score on the WebNLG dataset increases from 0.918 to 0.927. Similarly, the F1-score on the NYT dataset is improved from 0.896 to 0.904, while that on the NYT11-HRL dataset also rises from 0.539 to 0.550. The experiments indicate that using the Focal Loss function in the tail entity decoder of the CasRelBLC model successfully addresses the issue of sample imbalance and leads to notable enhancements concerning extraction performance. Regarding the efficacy of distant supervised noise reduction, the use of reinforcement learning to mitigate noise in the dataset NYT11-HRL results in additional enhancement of the model training effect that F1-score on the test set is improved from 0.550 to 0.561, which suggests that integrating the deep reinforcement learning noise reduction module yields significant noise reduction in distant supervised datasets, such that the performance of CasRelBLCF can achieve improvement. Compared with the latest baselines, our model achieves a 0.4% improvement in precision on the NYT dataset and a 2% improvement on the WebNLG dataset, with an overall F1-Score improvement of 0.9%. On the NYT dataset, our model exhibits the best average performance across the three metrics at 92.7%. This suggests that our approach effectively addresses the impact of sample imbalance and pays better attention to the continuity of sentences in head entity decoding on the extraction capability of ternary relation, further enhancing the model’s performance in extracting relational triplets from unstructured text.

In further research, we will investigate methods to enhance the decoding efficacy while maintaining the decoding speed of the head entity. Furthermore, this research only considers the false positive noise in the distant supervised dataset while designing the noise reduction module. However, it is essential to acknowledge that there is also a portion of false negative noise in the distant supervised dataset, which can still impact the effect of model training. Subsequent investigations may endeavor to identify methodologies aimed at mitigating the occurrence of misleading harmful noise.
